# Editorial for the Special Issue “Emerging Viral Zoonoses, Second Edition”

**DOI:** 10.3390/microorganisms12081662

**Published:** 2024-08-13

**Authors:** Jelena Prpić

**Affiliations:** Croatian Veterinary Institute, Savska Cesta 143, 10000 Zagreb, Croatia; balatinec@veinst.hr

The “One World–One Health” framework has underscored the critical need for interdisciplinary collaboration in understanding and combating emerging viral zoonoses [1]. These diseases, which bridge the animal–human–ecosystems interface, pose significant public health challenges worldwide.

Over 70% of pathogens that infect humans are zoonotic and viral in nature [2]. Zoonoses involves the transmission of pathogens from vertebrate animals (such as reptiles, mammals, and birds) to humans [3]. These animals act as reservoirs or amplifier hosts, facilitating the initial jump from animals to humans through direct or indirect interactions [4]. While viral zoonoses recur, humans often serve as dead-end hosts for these pathogens [5]. In rare cases, animal viruses can adapt so effectively to human hosts that they become host-exclusive [6]. Most viruses cannot coexist with humans and are shed from the blood, as well as the gastrointestinal, urogenital, and respiratory tracts without causing significant harm [6]. Acute viral zoonoses typically require constant reintroduction from non-human hosts to initiate human-to-human transmission [7].

The emergence and re-emergence of viral zoonoses have become more frequent in recent decades.

These zoonotic pathogens, which can jump from animals to humans, are influenced by various human activities. Factors such as deforestation, changes in farming practices, population dynamics, and societal shifts contribute to their spread [8]. Additionally, our impact on natural ecosystems plays a role in these outbreaks. By understanding these dynamics and addressing their root causes, we can mitigate the risk of future zoonotic disease outbreaks, thus improving public health [9].

Understanding the clinical manifestations of zoonotic infections is essential for their timely diagnosis and effective management. From tick-borne encephalitis virus to rabies virus, each pathogen presents unique challenges. Clinicians must recognize the subtle signs and symptoms, enabling early intervention and improved outcomes. Emphasizing prevention as the primary defense, innovative approaches like artificial intelligence are explored to predict outbreaks and guide preventive efforts. Robust surveillance systems, vector control, and vaccination campaigns are crucial. Addressing risk factors at the human–animal–environment interface helps to mitigate the emergence of new zoonoses. Once zoonotic infections occur, effective treatment becomes paramount. Collaborative efforts between human and veterinary medicine are vital to optimize treatment regimens and enhance patient outcomes. Advancements in molecular techniques allow us to dissect zoonotic viruses’ genetic makeup. Tracing their origins, transmission routes, and adaptations provides insights into their behavior. This knowledge informs both targeted interventions and global health policies.

Given the lack of acquired immunity in humans against emerging viral zoonotic diseases, as well as the loss of herd immunity against re-emerging ones, it is crucial to focus on zoonotic detection, prevention, and response. These diseases often result from interconnected anthropogenic factors. To address this, multisectoral initiatives, such as One Health partnerships, remain essential. The concept of One Health, which integrates human, animal, and environmental health, has gained renewed importance due to evolving interactions between animals, people, and plants. To ensure its success and global governance, we must overcome competitive interests between the public and private sectors. Additionally, bridging the gap between negative social perceptions and scientific data is vital, as social factors significantly impact disease transmission.

The battle against emerging viral zoonoses requires interdisciplinary collaboration, data sharing, and a commitment to One Health principles. As we navigate this complex landscape, we should remain vigilant, proactive, and united in safeguarding our shared well-being.
